# Heavy metals in children's blood from the rural region of Popokabaka, Democratic Republic of Congo: a cross-sectional study and spatial analysis

**DOI:** 10.1038/s41598-022-23332-4

**Published:** 2022-11-03

**Authors:** Branly Kilola Mbunga, Elin L. F. Gjengedal, Freddy Bangelesa, Mina M. Langfjord, Marc M. Bosonkie, Tor A. Strand, Mala Ali Mapatano, Ingunn M. S. Engebretsen

**Affiliations:** 1 Kinshasa School of Public Health, Faculty of Medicine, University of Kinshasa, Kinshasa, Democratic Republic of Congo; 2 Faculty of Environmental Sciences and Natural Resource Management, Norwegian University of Life Sciences, 1432 Ås, Norway; 3 Institute of Geography and Geology, University of Würzburg, Am Hubland, 97074 Würzburg, Germany; 4 Centre for International Health, Department of Global Public Health and Primary Care, University of Bergen, 5009 Bergen, Norway; 5 Department of Research, Innlandet Hospital Trust, 2609 Lillehammer, Norway

**Keywords:** Biochemistry, Environmental sciences

## Abstract

Exposure to heavy metals can affect cell differentiation, neurocognitive development, and growth during early life, even in low doses. Little is known about heavy metal exposure and its relationship with nutrition outcomes in non-mining rural environments. We carried out a community-based cross-sectional study to describe the distribution of four heavy metal concentrations [arsenic (As), cadmium (Cd), lead (Pb), and mercury (Hg)] in the serum of a representative population of children aged 12 to 59 months old from the rural region of Popokabaka, Democratic Republic of Congo. The four metals were measured in 412 samples using inductively coupled plasma–mass spectrometry (ICP–MS). Limits of detection (LoD) and quantification (LoQ) were set. Percentiles were reported. Statistical and geospatial bivariate analyses were performed to identify relationships with other nutrition outcomes. Arsenic was quantified in 59.7%, while Cd, Hg, and Pb were quantified in less than 10%, all without toxicities. The arsenic level was negatively associated with the zinc level, while the Hg level was positively associated with the selenium level. This common detection of As in children of Popokabaka requires attention, and urgent drinking water exploration and intervention for the profit of the Popokabaka community should be considered.

## Introduction

Regardless of multiple controversial definitions^1^, heavy metals (HMs) are commonly characterized as chemical elements with relatively high atomic numbers and densities (more than 20 and more than 5 g/cm^3^, respectively) that are naturally found on Earth and that vary geographically from one region to another^2^. In a place with a high concentration of HMs, they easily enter the food systems and contaminate all food products (including water) from the Earth in a cycling chain that could cause harmful effects on consumers^3,4^. These metals are of public health interest because of their direct harmful effects on human health^5,6^ and indirect interactions with other essential minerals^7^. They may cause poisoning and serious irreversible health effects even at low doses^8–11^. During pregnancy, exposure to heavy metals can be neurotoxic and may impair child development^12^. During infancy, additional routes of exposure, including breastfeeding and high-risk behaviors, such as hand-to-mouth activities, make children vulnerable to HM poisoning^13,14^.

Among the various HMs, arsenic (*As*), cadmium (*Cd*), lead (*Pb*), and mercury (*Hg*) are the most common and are considered harmful^9–11^. Arsenic is widely distributed in natural waters, and groundwater is one of the primary routes of exposure to inorganic *As*^15^. Long-term exposure to inorganic *As* during infancy increases the risk of lower respiratory tract infection, gastrointestinal illnesses, and cancer^15,16^. Lead, which has no physiological role in humans, is frequently found in household dust. More than 95% of the total *Pb* exposure ends up in the bones and teeth. Children are particularly vulnerable to lead because of its effects on growth and the developing nervous system^17^. Cadmium is mainly absorbed from the lungs, and tobacco smoke is one of the largest single sources of *Cd* exposure in humans^18^. It can accumulate in fatty tissues and human milk and be transferred through breast milk to children. Cadmium toxicity negatively affects reproduction, neurodevelopment, and hepatic, hematological, and immunological systems^10,18^. Mercury is used in agriculture in fungicides or seed preservatives and pharmaceutical catalysts in organic syntheses. Higher levels of *Hg* are often found in seafood, and exposure to this element is also suspected to impair neurodevelopment in children^12^ and cause dental, skin, pulmonary, and nephrotic damage^19^.

Although the global and nationwide prevalence and burden of HMs are not available, many regional studies^20–26^ have estimated the risk of heavy metal exposure in humans. In the Democratic Republic of Congo (DRC), little is known about the risk of heavy metal exposure in children and its spatial distribution. In the non-mining urban region of Kinshasa, Tuakila et al.^27^ reported *As* toxicity (95%), *Pb* toxicity (35%), and *Hg* toxicity (10%) in a sample of 100 children in 2014. In the same region, Ngweme et al.^28^, in 2021, recently alerted on the toxic detection in leafy marketable vegetables. From the urban mining region of Lubumbashi, Musimwa et al.^29^ reported antinomy (*Sb*)*, Pb*, and cobalt (*Co)* toxicities in children admitted to a nutrition rehabilitation center. While industrial and anthropogenic pollution can be seen as the most important sources of environmental pollution in many *urban* cities, the heavy metal spectrum from regular non-mining rural communities has not yet been established, including in the DRC. The limitation of these studies is that the spatial distribution and analysis of HMs are not addressed. The spatial aspect is particularly important because it contextualizes HMs to local environmental conditions (water sources, elevation, land cover, and land use), which may affect their distribution. Therefore, combining heavy metal assessment at both the individual and geospatial levels may facilitate understanding the potential geogenic or anthropogenic sources across the region and prepare for ecological health risk intervention patterns. We assessed serum *As, Pb, Hg, and Cd* levels in a community-based representative sample of children under the age of five from Popokabaka, DRC, and searched for relationships with nutrition, health and geospatial characteristics.

## Results

### Characteristics of the study participants

The study population characteristics have been published previously^30,31^. In short, the median age of the children was 32 months (Table 1). Approximately half (51.5%) of the children were boys, and 55.1% lived within households using groundwater sources for drinking water. Nearly 55% of children were stunted, and more than half (58%) experienced fever within the two preceding weeks of our visit. Approximately one-third (32.2%) reported coughing in the same period. Table 1 also summarizes the burden of essential mineral deficiencies among children in Popokabaka: selenium (Se) deficiency and zinc (Zn) deficiency were highly prevalent in the Popokabaka child population, at 86.9% and 64.6%, respectively.

**Table 1:** General characteristics of the study participants in Popokabaka.

Characteristics	N (412)	%
**Sociodemographic**		
Age as Median (Interquartile range)	32 (20.5)	
Sex-male	212	51.5
Drinking groundwater	238	55.1
Drinking water from rivers	174	44.9
**Anthropometric**		
Stunting	228	55.3
Wasting	44	10.7
Underweight	140	34.0
**Clinical**		
Fever in the two last weeks	239	58.0
Diarrhoea in the two last weeks	71	17.2
Cough in the two last weeks	133	32.3
**Biochemical**		
Anaemia	280	68.0
Inflammation state (elevated CRP)	202	49.0
Iron deficiency	53	12.9
Iron deficiency anaemia	31	7.5
Zinc deficiency	266	64.6
Selenium deficiency	358	86.9
Cooper deficiency	6	1.5

Table 2 describes the food frequency consumption of children over a recall period of seven days. The results from this table reveal that the regular diet is composed of starchy foods and green leaves. Fish and seafood, known to carry large amounts of minerals from rivers and oceans, were poorly consumed among the children of Popokabaka. Animal source foods such as meat, chicken, eggs, milk, and dairy products were rarely consumed. Sugar and palm oil are frequently used as food additives in this population.

**Table 2:** Food consumption characteristics are expressed as the number of days on a week scale the child consumed at least one item of the food groups.

Food groups	Median	p25	p75
Cereals	1	0	5
Vitamin A-rich leaves or tubers	0	0	2
Starch roots & tubers	7	7	7
Green leaves	4	3	5
Vitamin A rich fruits	0	0	1
Other Vegetables	2	1	4
Offal	0	0	0
Meat/chicken	1	0	2
Eggs	0	0	1
Fish and seafood	2	1	4
Vegetable oil	2	1	4
Milk/dairy products	0	0	0
Fatty foods	0	0	1
Sugar/sweeteners products	5	2	7
Coffee, tea, other stimulants	3	0	7
Insects	0	0	2
Palm oil	7	5	7

### Distribution of heavy metals

The results showed that *As, Hg, Cd,* and *Pb* were detected in 95.6%, 66.0%, 19.4%, and 13.1% of the samples, respectively (Table 3). More than half of children (59.7%) had quantifiable arsenic values, while *Hg, Cd,* and *Pb* were only quantified in less than 10% of children without any toxicity level.

**Table 3:** Prevalence of detection and quantification of heavy metals in Popokabaka Children, n (%) = 412 Children

	Undetected < LOD	Detected		
		No quantified LOD-LOQ	Quantified ≥ LOQ	Toxic
*As*	18 (4.4)	148 (35.9)	246 (59.7)	0 (0.0)
*Hg*	140 (34.0)	238 (57.8)	34 (8.2)	0 (0.0)
*Cd*	332 (80.6)	76 (18.5)	4 (0.9)	0 (0.0)
*Pb*	358 (86.9)	38 (9.2)	16 (3.9)	0 (0.0)

LOD = Limit of detection, LOQ = Limit of Quantification

Table 4 shows that the distribution was left-censored because of nonquantifiable serum values. Except for *As*, quantifiable values were distributed above the 95th percentile for *Hg, Cd,* and *Pb*.

**Table 4:** Percentile value distribution of heavy metals in Popokabaka Children (μg/L).

	Percentiles values Distribution (μg/L)					
	P5	P25	Me	P75	P95	P99
*As*	< LOD	LOD-LOQ	1.88	5.67	6.12	7.70
*Pb*	< LOD	< LOD	< LOD	LOD-LOQ	12.8	13.3
*Hg*	< LOD	< LOD	< LOD	LOD-LOQ	1.5	1.8
*Cd*	< LOD	< LOD	< LOD	LOD-LOQ	0.09	0.11

LoD for As 0.2 μg/L, LoQ for As 0.55 μg/L; LoD for Pb 0.6 μg/L, LoQ for Pb 2.1 μg/L;

LoD for Hg 0.2 μg/L, LoQ for Hg 0.83 μg/L; LoD for Cd 0.006 μg/L, LoQ for Cd 0.019 μg/L.

### Interaction between the detection of heavy metals and other nutrition outcomes

The non-parametric Kruskal Wallis test^32^ identified two statistically significant relationships (Table 5): first, *As* detection was negatively associated with *Zn* levels. Higher *As* levels were found in Zn-deficient children. Second, *Hg* detection was positively associated with Se levels in Popokabaka children. Children with higher *Hg* levels also had higher *Se* levels. No differences were found concerning anthropometry and growth across HM levels.

**Table 5:** Differences in the Median (interquantile range) level of some nutrition indicators across the HM levels using the Kruskal wallis test.

	Freq	HAZ	WHZ	WAZ	Se (µg/L)	Zn (µg/dL)	Cu (µg/dL)	Hb (g/dL)
**As_bin**								
< LOD	18	−2.1 (2.7)	−0.2 (1.2)	−1.3 (1.4)	49.5 (19.0)	65.1 (9.9)	140.5 (55.5)	10.1 (2.8)
[LOD-LOQ]	148	−2.4 (2.1)	−0.5 (1.1)	−1.6 (1.8)	53.7 (22.0)	64.1 (20.1)	146.0 (49.0)	10.3 (1.8)
≥ LOQ	246	−2.1 (1.8)	−0.5 (1.4)	−1.5 (1.4)	52.7 (18.9)	59.9 (15.8)	145.5 (47.0)	10.4 (1.7)
*P value*		0.49	0.87	0.57	0.30	0.002*	0.37	0.94
**Hg_bin**								
< LOD	140	−2.4 (2.3)	−0.5 (1.6)	−1.7 (1.6)	48.7 (17.0)	60.6 (16.5)	135.5 (49.5)	10.5 (1.5)
[LOD−LOQ]	238	−2.1 (1.9)	−0.5 (1.4)	−1.5 (1.6)	53.5 (18.0)	61.9 (18.5)	148.5 (40.0)	10.2 (1.9)
≥ LOQ	34	−2.2 (1.3)	−0.6 (1.6)	−1.5 (0.9)	67.3 (16.0)	64.0 (14.5)	162.5 (57.0)	10.5 (1.5)
*P value*		0.18	0.83	0.35	< 0.001*	0.25	0.046*	0.34
**Pb_bin**								
< LOD	358	−2.1 (2.1)	−0.5 (1.5)	−1.5 (1.7)	52.7 (20.0)	61.6 (16.6)	145.0 (46.0)	10.4 (1.9)
[LOD-LOQ]	38	−2.3 (1.6)	−0.6 (1.9)	−1.8 (1.7)	53.9 (13.6)	65.9 (19.6)	154.0 (53.0)	10.3 (1.9)
≥ LOQ	16	−2.1 (1.7)	−0.2 (1.2)	−1.4 (1.3)	48.9 (18.0)	63.7 (19.5)	137.5 (51.0)	10.5 (1.6)
*P value*		0.81	0.54	0.46	0.52	0.42	0.55	0.83
**Cd_bin**								
< LOD	332	−2.1 (2.1)	−0.5 (1.0)	−1.5 (1.6)	52.6 (20.8)	62.2 (17.0)	147.0 (45.0)	10.4 (1.9)
[LOD-LOQ]	76	−2.4 (2.0)	−0.7 (1.3)	−1.7 (1.5)	54.3 (14.5)	59.4 (18.3)	136.5 (41.0)	10.3 (1.6)
≥ LOQ	4	−1.9 (1.7)	−0.6 (0.7)	−1.3 (1.3)	48.4 (42.2)	66.4 (23.1)	130.5 (105)	10.3 (0.3)
*P value*		0.42	0.42	0.17	0.69	0.88	0.13	0.98

* means significant pvalue.

HAZ = height-for-age Zscore; WHZ = weight-for-height Zscore; WAZ = weight-for-age Zscore;

Se = selenium; Zn = Zinc; Cu = copper; Hb = haemoglobin.

### Spatial variation in As/Zn and Hg/Se interactions

**Figure 1:**
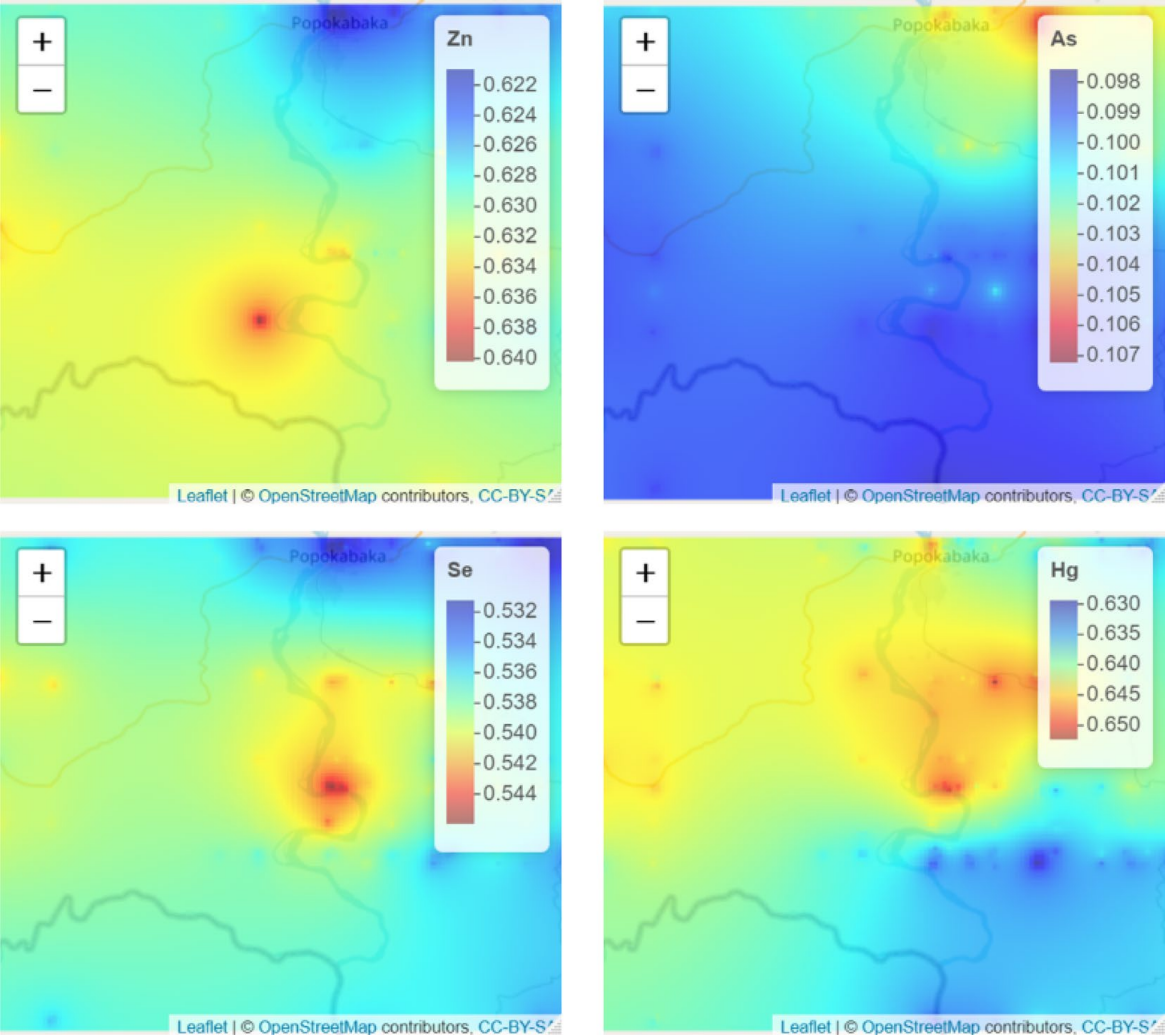
Spatial distribution and variation in As, Zn, Se, and Hg in Popokabaka. For plotting purposes, the concentrations of Se, As, and Hg were multiplied by 10, 100 and 1000, respectively. The inverse distance's weighting is based on the optimum power obtained after the cross-validation.

**Figure 2:**
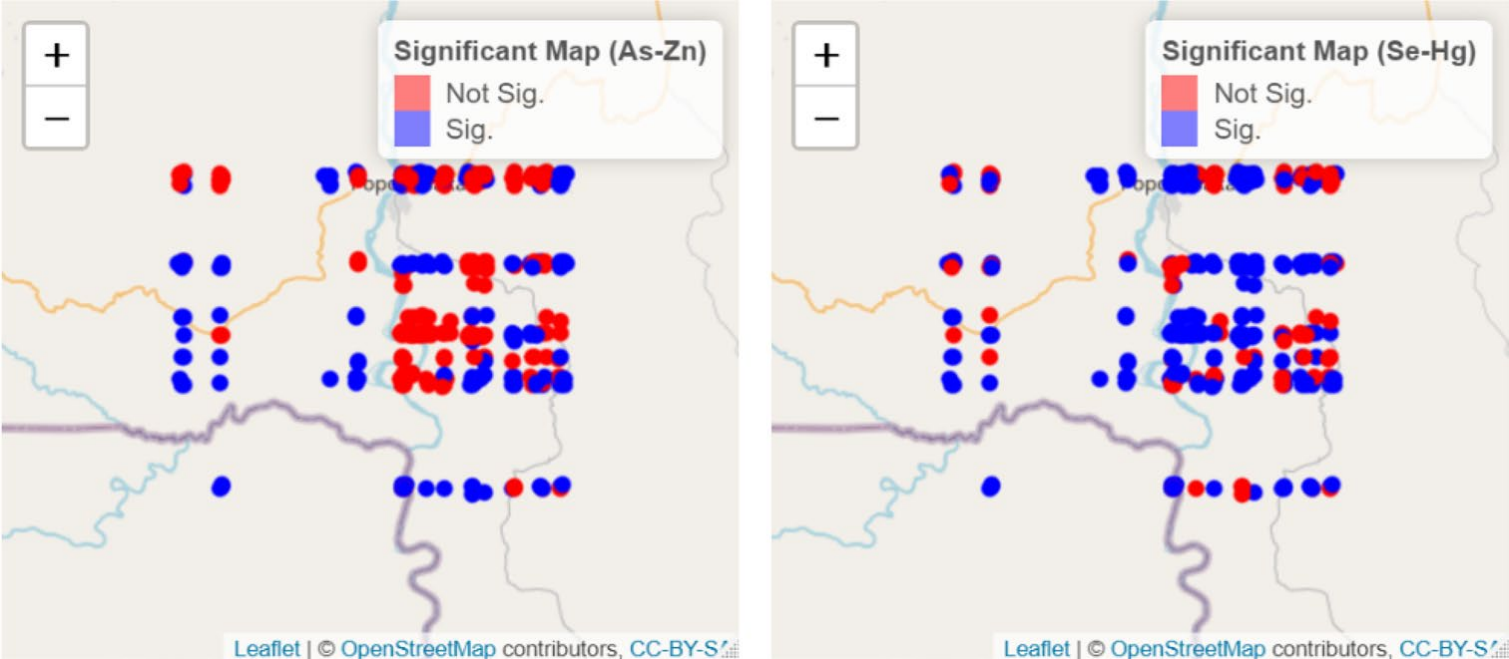
Local bivariate spatial association between As and Zn, and Se and Hg. Blue dots indicate a significant spatial association, and red dots indicate a nonsignificant spatial association. Geographic coordinates were systematically moved to a given direction (200 m) to avoid identifying specific households. This displacement did not affect the spatial relation of points.

The spatial distribution maps of *As, Zn, Se,* and *Hg* are presented in Fig. 1. The spatial pattern indicates a low Zn concentration in the northern part of the study region, where *As* is more concentrated. In addition, the spatial distribution of *Se* and *Hg* is almost the same, with a high concentration around the Kwango River. The spatial distribution (Fig. 1), using the global bivariate spatial association index, showed a significant spatial dependency between Hg and Se (L = 0.12, *P* value < 0.001), implying that high concentrations of Hg were spatially associated with high concentrations of Se. A significant spatial discrepancy was observed between *As* and *Zn* (L =− 0.07, *P* value < 0.001). The local bivariate spatial association is presented in Fig. 2, indicating the local contribution of observation to the spatial association. The blue color for the Se–Hg association indicates households where a high concentration of *Se* is significantly associated with high concentrations of Hg. In contrast, for the *As-Zn* association, the same color indicated areas where high values of *Zn* were significantly associated with low values of *As*, thus suggesting a lack of local association.

## Discussion

In the present community-based study of children of Popokabaka, we screened for the existence and distribution of the four most significant potential HMs^33^. Arsenic was detected in almost all children (95.6%), of which more than half (59.7%) had quantifiable values. Mercury was detected in 66.0% of children, with fewer (8.2%) having quantified values. Lead and cadmium had relatively low detection rates of 13.1% and 19.4%, respectively. Arsenic was negatively related to Zn, while mercury was positively related to selenium levels, both statistically and geographically. These findings suggest environmental exposures.

Since the arsenicosis crisis report from Bangladesh^34^ due to *As* contamination in drinking water, there has been an increasing public health interest in this metal. Recently, two papers^35,36^ reported that in Africa, *As* is spatially abundant in water, soil, sediment, fish, and vegetation and advocated for human exposure and health effect assessment. Similarly, our results suggest that *As* is widespread in Popokabaka, and communities could be exposed to a permanent source. For example, as in any rural context, untreated contaminated water used for drinking, cooking, and irrigating crops may be an important source. Consumption of contaminated seafood could also result in elevated *As* concentrations. There is compelling evidence that consumption of predatory fish such as shellfish, sea mammals, and other (shark, swordfish, mackerel, tilefish from the ocean) increase the As level^37^. However, as shown in Table 2, fish and seafood consumption in Popokabaka is relatively low. The geographical inaccessibility of this area also excludes any imported sources of such foods. Arsenic exists in different forms in nature: toxic in acute and poisoning conditions (arsenate, arsenite), nontoxic when metabolized by the body (monomethyl-arsine, dimethyl-arsine), and nontoxic in food (arsenobetaine, arsenocholine). Inorganic *As* is the most lethal and carcinogenic^37^. In this study, we reported the total As concentration and additional analysis is required to specify the form.

Geospatial analysis showed that the highest exposure to *As* in children north of Popokabaka and on the eastern side of the Kwango River. This also implies that the presence of *As* varies in the soil of Popokabaka. In addition, the arsenic was more commonly found in children with more profound Zn deficiency. This interaction between As and Zn is supported in the literature. Kader et al.^38^ who reported that presence of the two minerals in the soil could lead to chelation. The authors concluded that Zn uptake in plants was significantly reduced in As-containing soils.

Lead, Hg and Cd usually share the same geogenic or industrial sources and have been more frequently reported from mining or rural regions^24,26–28,39,40^. They leach from geogenic granite rock or industrial pollution and enter the food chain by contamination. They are well-known polluants metal from the environment^33^. Contrarily to arsenic, they are highly toxic, unnecessary for human metabolism, and cause, at a low level, severe damage to the nervous system, development, and behavioral performance^8^. Our data has revealed that the 95th percentiles of Pb, Hg, and Cd were respectively at 12.8 μg/L, 1.5 μg/L, and 0.09 μg/L. No toxic level has been quantified among children, but the proportion detected was quite significant, as 66% for Hg, 19.4% for Cd, and 13.1% for Pb. This high proportion of detection of low Hg levels is of concern and should be a prioritized research question. The literature supports that a diet favoring seafood is associated with a high level of blood Hg^41^***.*** Fish consumption can explain most of the blood Hg in Popokabaka. Even if overall fish consumption is low in Popokabaka, it may likely be higher along the river than in other areas. Also, fish consumption can explain the positive association (statistical and geographic) between mercury and selenium. Both elements are common in fish, and the area of high detection/exposure is along the river. This environmental source and others should be of priority interest in further exploration.

The literature supports inverse relationships between these three metals and children's IQ and growth. Our study found no relationship between the growth and detection of HMs. The Lancet^42^ pointed out that co-exposure to multiple metals increases neurotoxicity and leads to a decline in neurocognitive development during early life.

We assessed the four HMs in blood under fasting conditions (> 8 h from the last meals). Another study^43^ reported that these four metals have blood concentrations elevated only for a short time after ingestion (4–6 h). They are rapidly metabolized by the liver, accumulate in specific tissues (keratins), and are excreted by the kidney. Considering that, the HM levels we reported could be underestimated^43^. In addition, It was also impossible to capture the chronic exposure and accumulation history. Studies that use matrices such as nails, hair, and urine could complement and improve our understanding. This study revealed a high proportion of value < LoD or < LoQ. These censored data do not simply mean zero value but are undetected with the highly sensitive ICP-MS we have used. In this context, further environmental exploration to assess the risk of exposures for this category. The selection of a spatial interpolation method may impact the distribution map of HMs and their associated minerals. Sophisticated spatial interpolation methods such as kriging could have been applied because they provide the best linear unbiased estimates and highlight local variations^44^. However, it was very complex to fit the semi-variogram, probably due to the distribution of sample points (rand cluster). In some circumstances, the inverse distance seemed to suit this study and its outcomes kriging^44^. In addition, the result of the bivariate spatial association could also have been affected by the number of neighborhoods, which was set to four (standard) in this study.

Despite these limitations, we have reported a picture of heavy metals in a representative rural community for the first time in DRC. Most of the studies are hospital-based and low-scale. The lab analysis method used in this study, ICP–MS, is the most accurate and is indicated in the study of trace elements. Statistical analysis was confirmed and completed by advanced geospatial techniques to better describe the distribution and variability of these HMs.

## Conclusions

The high occurrence of As and other HM detection reported in this study implies that Popokabaka should be considered an area with certain HM hazards. Based on existing data, we suggest a geogenic source (soil) and the ingestion of contaminated food and drinking water as possible pathways. However, biomonitoring and deeper exploration are needed to establish environmental causal pathways that could help adapt defensive measures to prevent health and nutrition damage in communities.

## Methods

### Study design and location

**Figure 3:**
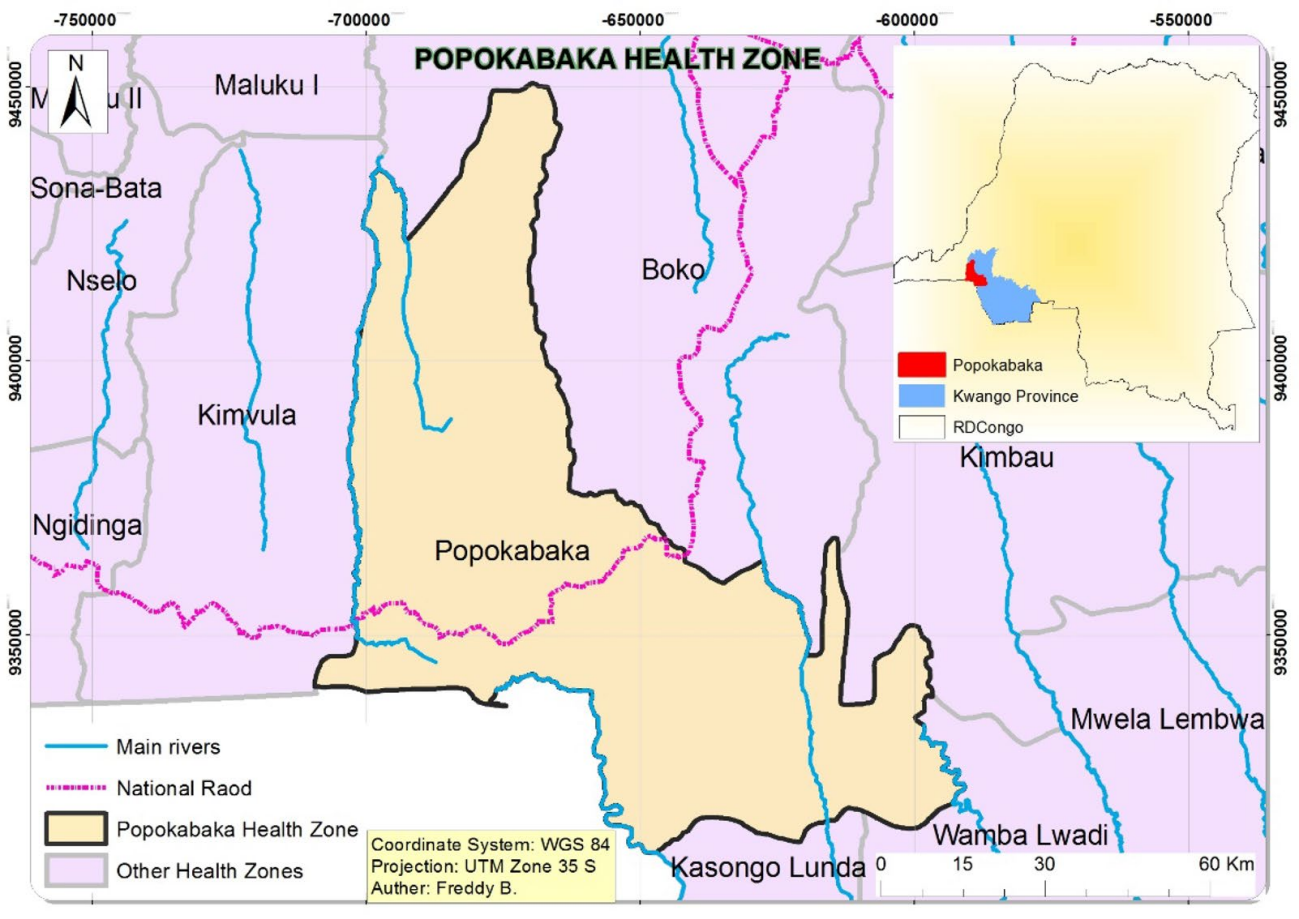
Location of Popokabaka Health Zone. Source: shapefile downloaded from the Humaniterian Data Exchange (https://data.humdata.org/dataset/drc-health-data) and map created by Freddy Bangelesa using ArcGIS 10.4, 2022".

We conducted a community-based cross-sectional study in the Popokabaka Health zone, Kwango Province, DRC, between May and June 2019. The region (5°3,803,500–5°430,000 latitude South, 16°3,406,000–16°370,800 longitude East) is entirely rural without any known mining history (see Fig. 3). It is close to the Kahemba region and the Angola country border, two areas known for diamond mining exploitation. The Kwango River that crosses the Popokabaka region takes its source and crosses these mining regions. Agriculture, which is not varied, has cassava as the principal plant grown. The use of fertilizers is not common among farmers. Konzo, a neurotoxic motor disease, is also prevalent in the region^45–48^. People drink untreated water from diverse groundwater sources. Families live under a house built on earth materials. Growth retardation is severe: one in two children is stunted^49,50^. Information on congenital malformation and cancer is not available.

### Participants and sampling

As the present research is part of a multiple-outcomes biomarker survey, the minimum sample size was based on a proportional calculation for anemia prevalence of 0.59, a precision of 0.075, a design effect of two, and a response rate of 0.80. The total size of 412 children aged 12–59 months was considered in this study. Children were selected using a three-stage cluster sampling technique. More details on sampling are described in our former article^31^. They belonged to 5 clusters known as health areas which were selected as part of the cluster sampling procedure. Those were the Kabangu, Ingasi, Cite-Popo, Secteur-Popo, and Tzunza health areas.

### Data collection and blood processing in the field

Data were collected using a questionnaire completed on android tablets using the Survey CTO application. The digital questionnaire consisted of eight modules: household characteristics; water, hygiene, and sanitation (WASH); household food security (Household Food Insecurity Access Scale-HFIAS); child health history; Infant Feeding practices; anthropometric measures; dietary patterns (24 h recall and food frequency); and biochemical sampling. Household geocoordinates (longitude, latitude, altitude, and precision) were captured for children's location using the “geopoint” command in the digital questionnaire. Data collection was organized on two consecutive days in each cluster: a household survey with anthropometry on the first day and blood collection on the following day. More details can be found in our previous papers^30,31^.

### Blood processing and management

We used serum BD vacutainers (BD-368380), trace-element-free equipment, powder-free sterile disposable gloves, plastic surfaces, and techniques previously described^31^ to ensure that samples were not contaminated. We collected up to 6 mL of venous blood from each child and separated the serum from the blood cells within 3 h^30,31^. Separation was performed at 2300 rpm for 10 min (RCF 1532 g) using a Hettich centrifuge (Tuttlingen, Germany). Serum was aliquoted into two 2-ml polypropylene vials (Sarstedt, Nümbrecht, Germany). Vials were stored at –40 °C while completing the fieldwork and then transported from the Popokabaka area to Kinshasa (12 h of vehicle trip) and stored in liquid nitrogen. There, vials were stored at −80 °C at the Kinshasa School of Public Health for a week before being shipped with dry ice to the Norwegian University of Life Sciences’ Laboratory (Aas, Norway) for analysis.

### Laboratory processing and assessment

#### Sample preparation

Using a 100- to 1000-µL pipette (Sartorius, Göttingen, Germany) and Thermo Scientific ART Barrier pipette tips (Waltham, MA, USA), 250-µL aliquots of thawed, tempered, and homogenized serum were transferred into 5-mL polypropylene tubes (Sarstedt, Nümbrecht, Germany) and accurately weighed (Sartorius MC 210P). Subsequently, using a 10–300 µL electronic pipette (Biohit, Helsinki, Finland), 100 µL of internal standard (rhodium (Rh) and selenium (^74^Se)) and 500 µL nitric acid (HNO_3_, 69% weight (w)/w, sub-boiled ultra-pure) were added to each sample before digestion for three hours at 90 °C in a heating cabinet (Termaks, Bergen, Norge). Furthermore, the samples were quantitatively transferred into polypropylene centrifuge tubes (Sarstedt, Nümbrecht, Germany) and finally diluted to 500 mL with deionized water (> 18 MΩ). To stabilize mercury in the solution, 100 µL hydrochloric acid (HCl, 37% w/w, sub-boiled ultra-pure) was added to each sample.

#### Analysis of samples

Quantification of the total element concentrations in serum was conducted by inductively coupled plasma–mass spectrometry using the Agilent 8900 Triple Quadrupole (QQQ) ICP–MS^51^. The masses were (Q1/Q2): *Pb* (sum of 206/206, 207/207, and 208/208) with gas mode ammonia (NH_3_), *Cd* (111/111), and *Hg* (202/202) using gas mode oxygen (O_2_). Detection limits (LoD) and quantification limits (LoQ) were standard deviations of the blank samples (n = 10) multiplied by three and ten, respectively. Blank samples were taken through the measurement procedure, including the sample preparation steps. The LoD/LoQ ratios (n = 10) were determined in mg/L as (0.0002/0.00055), *Cd* (0.000006/0.000019), *Hg* (0.0002/0.00083) and *Pb* (0.0006/0.0021).

#### Quality control

We assessed blank samples for contamination of reagents and equipment used. Accuracy was checked by concurrent analysis of Seronorm™ TEs Serum L1 and L2 (Billingstad, Norway). The obtained data for Hg were within a 95% confidence level of the certified values issued. Compared with analytical values issued, bias equal to 1.5% (L1) and 1.7% (L2) for Cd and 5.0% (L1) and 9.4% (L2) for Hg was noticed, while with respect to Pb in Serum L1 and L2, the results were < LoQ. Despite low levels of As (< LoQ), compared with the analytical values issued, a bias equal to 22% (L1) and 21% (L2) was revealed. The method's within-laboratory reproducibility (RSD) was 12% for *Cd,* while As, *Pb,* and *Hg* were inconclusive since obtained values were below LoQ; the results were obtained by carrying out measurements on 12 replicate samples aliquoted from a pooled sample of serum and measured on three different days. Method's repeatability (RSD) determined on Serum L1 (n = 5) and L2 (n = 5), were 13% and 8.6% with respect to Hg and 11% and 4.0% with respect to Cd. Repeatability of Pb determined by analysis of Serum L1 and L2 were inconclusive. However, regarding As, the repeatability was estimated to 16% (L1) and 17% (L2) calculated on results < LoQ. It is important to note that As was quantitatively determined in 59.6% of the 412 serum samples (Table 3). The repeatability for As is expected to improve for actual serum samples since the measurement uncertainty increases near the LoQ.

### Heavy metal thresholds

The upper limits of concentrations of *As* (< 20 μgL-1), *Pb* (< 50 μgL-1), *Hg* (< 50 μgL-1), and *Cd* (< 50 μgL-1) for any acute exposure in children were taken from Carl Burtis and David Bruns^37^.

### Other nutrition outcomes definition

Anthropometric indices included weight-for-height, height-for-age, weight-for-age, and mid-upper-arm-circumference-for-age, and their Z scores were calculated using WHO Anthro software^52^.

Wasting was defined as a weight-for-height Z-score (WHZ) <  − 2, stunting was defined as a height-for-age Z-score <  − 2, and underweight was defined as a weight-for-age Z-score <  − 2. Biochemical measures included Hgb, Cu, Zn, and Se. Anemia was defined as levels < 11 g/dL^14^, Iron Deficiency (ID) as Transferrin saturation is < 20%. Cu deficiency as Cu < 80 µg/dL, Zn deficiency as < 65 µg/dL^27^, and Se deficiency as < 7.0 µg/dL^10^.

### Statistical analysis

Data were analyzed using Stata 16.1 (StataCorp LLC, Texas, USA). First, we described the proportions and patterns of data below LoDs, between LoDs and LoQs, and above LoQs cut-offs. Parametric measures such as *means* could not be computed due to the high proportion (> 65%) of undetected/unquantified values (left-censored distribution)^53^. In this case, substitution by unique values and imputation model techniques might overestimate, bias, or fabricate data^54^. Instead, statistics were summarized using a non-parametric method suggested by Tekindal et al.^55^ and percentiles (5th, 50th, 75th, 95^th^, and 99th percentiles). The difference in continuous nutritional outcomes was checked across HMs category levels using the non-parametric Kruskal Wallis test^32^. A significant difference was indicated by *P* < 0.05.

### Spatial analysis

Spatial analysis was conducted in R (R Core Team, 2014), and maps were produced using the package leaflet. The spatial analysis concerned only heavy metals with a proven statistical association with essential minerals. This concerns As, Hg, Cu, and Se. We used the inverse distance weighted spatial interpolation approach to map the distribution of these minerals/heavy metals^44,56^. Spatial statistics were applied using Lee's L bivariate spatial autocorrelation test^57^ to capture the spatial association between heavy metals and other essential minerals. This test integrates information from Pearson's r (spatial bivariate association measure) and Moran's I (univariate spatial association measure). The weight matrix was defined using the k nearest neighbor approach, and the number of k was set to four^58^. The value of the global L index varies between −1 and 1. Both positive and negative values indicate spatial association—spatial dependency for positive values and spatial discrepancy for negative ones. A value of zero indicates that both variables are randomly distributed (no spatial association). A pseudo-significant test based on Monte Carlo simulation of a stochastic permutation process^39^ with 10 000 permutations was computed to measure the significance of the association. The test compares the observed pattern to the theoretical random pattern. The null hypothesis is that there is no spatial association, and the alternative is that there is a spatial association. We set the significant difference at *P* < 0.05. We further computed the local bivariate spatial association to assess the individual area's contribution to the global L and the spatial bivariate heterogeneity.

### Ethical considerations

The study was conducted according to the guidelines of the Declaration of Helsinki and approved by the Norwegian Institutional Review Board" REK. Committee (ref: 2018/1420/R.E.K. vest, date: 30.11.2018) and the Kinshasa School of Public Health ethical committee (ref: ESP/CE/2019, date: 28.01. 2019) and in Bergen. Other authorizations were requested from the local administrative and health authorities. Written informed consent was obtained from mothers or caretakers of children in this study. A systematic spatial displacement of 200 mm was applied to a given direction to avoid identifying concerned households.

## Data Availability

The dataset of this study can be made available on a reasonable request to BKM.

## References

[1] Ali H, Khan E (2018). What are heavy metals? Long-standing controversy over the scientific use of the term ‘heavy metals’–proposal of a comprehensive definition. Toxicol. Environ. Chem..

[2] Onakpa MM, Njan AA, Kalu OC (2018). A review of heavy metal contamination of food crops in Nigeria. Ann. Glob. Heal..

[3] Rai PK, Lee SS, Zhang M, Tsang YF, Kim KH (2019). Heavy metals in food crops: Health risks, fate, mechanisms, and management. Environ. Int..

[4] Hapke H-J (1996). Heavy metal transfer in the food chain to humans. Fertil. Environ..

[5] Rahman Z, Singh VP (2019). The relative impact of toxic heavy metals ( THMs ) ( arsenic ( As ), cadmium ( Cd ), chromium ( Cr )( VI ), mercury ( Hg ), and lead ( Pb )) on the total environment : an overview. Env. Monit Assess-Springer.

[6] Sall ML (2020). Toxic heavy metals : impact on the environment and human health, and treatment with conducting organic polymers, a review. Environ. Sci. Pollut. Res..

[7] Goyer RA (1997). Toxic and essential metal interactions. Annu. Rev. Nutr..

[8] Fu Z, Xi S (2020). The effects of heavy metals on human metabolism. Toxicol. Mech. Methods.

[9] Jaishankar M, Tseten T, Anbalagan N, Mathew BB, Beeregowda KN (2014). Toxicity, mechanism and health effects of some heavy metals. Interdiscip. Toxicol..

[10] Witkowska D, Słowik J, Chilicka K (2021). Heavy metals and human health: Possible exposure pathways and the competition for protein binding sites. Molecules.

[11] Morais S, Garcia F, Pereira MDL (2014). Heavy metals and human. Health.

[12] Dack K, Fell M, Taylor CM, Havdahl A, Lewis SJ (2022). Prenatal mercury exposure and neurodevelopment up to the age of 5 years: A systematic review. Int. J. Environ. Res. Public Health.

[13] Yang F, Massey IY (2019). Exposure routes and health effects of heavy metals on children. Biometals.

[14] WHO. *Children’s health and the environmentnt: a global perspective and a resource manual for the health sector*. (WHO Edition, 2010).

[15] Kuivenhoven, M. & Mason, K. *Arsenic toxicity*. (StatPearls Publishing, 2021). 31082169

[16] Abdul KSM, Jayasinghe SS, Chandana EP, Jayasumana C, De Silva PMC (2015). Arsenic and human health effects: A review. Environ. Toxicol. Pharmacol..

[17] Wani AL, Ara A, Usmani JA (2015). Lead toxicity : A review. Interdiscip. Toxicol..

[18] Rahimzadeh M, Rahimzadeh M, Kazemi S, Moghadamnia A (2017). Cadmium toxicity and treatment: An update. Casp. J Intern Med.

[19] Counter SA, Buchanan LH (2004). Mercury exposure in children: A review. Toxicol. Appl. Pharmacol..

[20] Rehman K, Sajid M, Akash H (2018). Prevalence of exposure of heavy metals and their impact on health consequences. J. Cell. Biochem. Biochem..

[21] Hessabi M (2019). Concentrations of lead, mercury, arsenic, cadmium, manganese, and aluminum in blood of romanian children suspected of having autism spectrum disorder. Int. J. Environ. Res. Public Health.

[22] Rahbar MH, Samms-vaughan M, Dickerson AS (2015). Concentration of lead, mercury, cadmium, aluminum, arsenic and manganese in umbilical cord blood of jamaican newborns. Int. J. Environ. Res. Public Health.

[23] Gomez MD, Sabra S, Malmqvist E, Saborit A, Grataco E (2017). Heavy metals exposure levels and their correlation with different clinical forms of fetal growth restriction. PLoS ONE.

[24] Rahbar MH (2021). Concentrations of lead, mercury, arsenic, cadmium, manganese, and aluminum in the blood of pakistani children with and without autism spectrum disorder and their associated factors. Int. J. Environ. Res. Public Health.

[25] Silver MK (2019). Distribution and predictors of 20 toxic and essential metals in the umbilical cord blood of Chinese newborns. Chemosphere.

[26] Eom S (2018). Lead, mercury, and cadmium exposure in the korean general population. J. Korean Med. Sci..

[27] Tuakuila J, Kabamba M, Mata H, Mata G (2014). Toxic and essential elements in children’s blood (<6 years) from Kinshasa, DRC (the Democratic Republic of Congo). J. Trace Elem. Med. Biol..

[28] Ngweme GN, Konde JNN, Laffite A (2021). Contamination levels of toxic metals in marketed at Kinshasa. Democratic Republic of the Congo..

[29] Musimwa AM, Kanteng GW, Kitoko HT, Luboya ON (2016). Trace elements in serum of malnourished and well-nourished children living in Lubumbashi and Kawama Aimée. Pan Afr. Med. J..

[30] Mbunga BK (2022). Distribution and determinants of serum zinc, copper, and selenium levels among children under five years from Popokabaka, Democratic Republic of Congo: A cross-sectional study. Nutrients.

[31] Mbunga BK (2021). Prevalence of anemia, iron-deficiency anemia, and associated factors among children aged 1–5 years in the rural, malaria-endemic setting of Popokabaka, Democratic Republic of Congo: A cross-sectional study. Nutrients.

[32] Theodorsson-Norheim E (1986). Kruskal-Wallis test: BASIC computer program to perform nonparametric one-way analysis of variance and multiple comparisons on ranks of several independent samples. Comput. Methods Programs Biomed..

[33] CDC. The ATSDR 2015 priority list of hazardous substances. (2020). Available at: https://www.atsdr.cdc.gov/spl/. Updated 2020. (Accessed: 1st October 2022)

[34] Ahmad A, Khan M, Haque M (2018). Arsenic contamination in groundwater in Bangladesh : Implications and challenges for healthcare policy. Risk Manag. Healthc. Policy.

[35] Irunde R, Ijumulana J, Ligate F, Maity JP, Ahmad A, Mtamba J, Bhattacharya P (2022). Arsenic in Africa: Potential sources, spatial variability, and the state of the art for arsenic removal using locally available materials. Groundw. Sustain. Dev..

[36] Ahoulé DG, Lalanne F, Mendret J, Brosillon S, Maiga AH (2015). Arsenic in African waters : A review. Water Air Soil Polution.

[37] Burtis, C. A. & Bruns, D. E. *Tietz Fundamentals of Clinical Chemistry and Molecular Diagnostics 7e*. (2015).

[38] Kader M, Lamb DT, Wang L, Megharaj M, Naidu R (2017). Zinc-arsenic interactions in soil: Solubility, toxicity and uptake. Chemosphere.

[39] Jiang C (2021). Distribution, source and health risk assessment based on the Monte Carlo method of heavy metals in shallow groundwater in an area affected by mining activities, China. Ecotoxicol. Environ. Saf..

[40] Tuakuila J, Lison D, Lantin A, Haufroid V, Hoet P (2012). Worrying exposure to trace elements in the population of Kinshasa, Democratic Republic of Congo ( DRC ) Worrying exposure to trace elements in the population of Kinshasa, Democratic Republic of Congo ( DRC ). Int. Arch. Occup. Environ. Health.

[41] Lee CC, Chang JW, Huang HY, Chen HL (2012). Factors influencing blood mercury levels of inhabitants living near fishing areas. Sci. Total Environ..

[42] Grandjean P, Landrigan PJ (2014). Neurobehavioural eff ects of developmental toxicity. Lancet Neurol..

[43] Haller W, Bines JE (2012). Starvation and fasting: Biochemical Aspects. Encycl. Hum. Nutr..

[44] Mueller TG (2004). Map quality for ordinary kriging and inverse distance weighted interpolation. Soil Sci. Soc. Am. J..

[45] Ngudi DD, Banea-mayambu J, Lambein F, Kolsteren P (2011). Konzo and dietary pattern in cassava-consuming populations of Popokabaka, Democratic Republic of Congo. Food Chem. Toxicol..

[46] Bradbury, J. H., Mandombi, C., Nahimana, D., Banea, J. P., Denton, I., & Kuwa, N. (2011) Control of konzo in the Democratic Republic of Congo. *Nat. Prec.* 1-1. 10.1016/j.fct.2013.08.012 23941775

[47] Banea JP, Bradbury JH, Mandombi C, Nahimana D, Denton IC, Foster MP, Katumbay DT (2015). Konzo prevention in six villages in the DRC and the dependence of konzo prevalence on cyanide intake and malnutrition. Toxicol. Rep..

[48] Nzwalo H, Cliff J (2011). Konzo: from poverty, cassava, and cyanogen intake to toxico-nutritional neurological disease. PLoS Negl. Trop. Dis..

[49] National Institute of Statistitics. *Multiple Indicators clustered Survey 2017 2018-Congo Demogratic*. *Survey Finding Report* (2017).

[50] MinisterePlan_DRC. *Congo democratic republic - Democratic health survey (DHS) 2013–2014*. (2014).

[51] Agilent. *Leave Interferences Behind With MS/MS: The Agilent 8900 Triple Quadrupole ICP-MS*. (Agilent Technologies, 2020).

[52] WHO. Child growth standards and WHO Anthro software tools. (2022). Available at: https://www.who.int/tools/child-growth-standards/software. (Accessed: 4th August 2022)

[53] Hessel, D. *Statistics for censored Environnemental data Using Minitab and R, 2nd Edition*. (2012).

[54] Canales RA, Wilson AM, Pearce-Walker JI, Verhougstraete MP, Reynolds KA (2018). Methods for handling left-censored data in quantitative microbial risk assessment. Appl. Environ. Microbiol..

[55] Tekindal MA, Erdoğan BD, Yavuz Y (2017). Evaluating left-censored data through substitution, parametric, semi-parametric, and nonparametric methods: A simulation study. Interdiscip. Sci. Comput. Life Sci..

[56] Conolly, J. Spatial Interpolation. in *Archaeological Spatial Analysis* 118–134 (2020). 10.4324/9781351243858-7

[57] Lee S (2001). Developing a bivariate spatial association measure: An integration of Pearson’s r and Moran’s I. J. Geogr. Syst..

[58] Gerkman LM, Ahlgren N (2014). Practical proposals for specifying k- nearest neighbours weights matrices. Spat. Econ. Anal..

